# IL-15 Superagonist Expands mCD8^+ ^T, NK and NKT Cells after Burn Injury but Fails to Improve Outcome during Burn Wound Infection

**DOI:** 10.1371/journal.pone.0148452

**Published:** 2016-02-09

**Authors:** Naeem K. Patil, Liming Luan, Julia K. Bohannon, Yin Guo, Antonio Hernandez, Benjamin Fensterheim, Edward R. Sherwood

**Affiliations:** 1 Department of Anesthesiology, Vanderbilt University Medical Center, Nashville, TN, United States of America; 2 Department of Pathology, Microbiology and Immunology, Vanderbilt University Medical Center, Nashville, TN, United States of America; Istituto Superiore di Sanità, ITALY

## Abstract

**Background:**

Severely burned patients are highly susceptible to opportunistic infections and sepsis, owing to the loss of the protective skin barrier and immunological dysfunction. Interleukin-15 (IL-15) belongs to the IL-2 family of common gamma chain cytokines and stimulates the proliferation and activation of T (specifically memory CD8), NK and NKT cells. It has been shown to preserve T cell function and improve survival during cecal ligation and puncture (CLP)-induced sepsis in mice. However, the therapeutic efficacy of IL-15 or IL-15 superagonist (SA) during infection after burn injury has not been evaluated. Moreover, very few, if any, studies have examined, in detail, the effect of burn injury and infection on the adaptive immune system. Thus, we examined the effect of burn and sepsis on adaptive immune cell populations and the effect of IL-15 SA treatment on the host response to infection.

**Methods:**

Mice were subjected to a 35% total body surface area burn, followed by wound infection with *Pseudomonas aeruginosa*. In some experiments, IL-15 SA was administered after burn injury, but before infection. Leukocytes in spleen, liver and peritoneal cavity were characterized using flow cytometry. Bacterial clearance, organ injury and survival were also assessed.

**Results:**

Burn wound infection led to a significant decline in total white blood cell and lymphocyte counts and induced organ injury and sepsis. Burn injury caused decline in CD4^+^ and CD8^+^ T cells in the spleen, which was worsened by infection. IL-15 treatment inhibited this decline and significantly increased cell numbers and activation, as determined by CD69 expression, of CD4^+^, CD8^+^, B, NK and NKT cells in the spleen and liver after burn injury. However, IL-15 SA treatment failed to prevent burn wound sepsis-induced loss of CD4^+^, CD8^+^, B, NK and NKT cells and failed to improve bacterial clearance and survival.

**Conclusion:**

Cutaneous burn injury and infection cause significant adaptive immune dysfunction. IL-15 SA does not augment host resistance to burn wound sepsis in mice despite inducing proliferation and activation of lymphocyte subsets.

## Introduction

Infection is a common cause of complications in severely burned patients. Burn injury causes disruption of the protective skin barrier. This offers an ideal opportunity for microbial colonization and possible dissemination into blood and other organs, which can lead to sepsis. Thus, infection is the leading cause of death among burn patients that survive the early burn shock phase. A clinical observational study by Williams *et al*. showed that sepsis accounted for 47% of death among burn patients at a major pediatric burn center [1]. Another study by Mann *et al*. revealed that burn patients have a high prevalence of sepsis and associated poor outcomes [2]. Further studies showed that burn size is directly proportional to the observed all-cause mortality among burn patients. Studies by Kraft *et al*., reported that a burn size of 60% is a crucial threshold for post burn morbidity and mortality [3]. However, many factors contribute to morbidity and mortality in burned patients. Recent papers indicate that elderly patients (>65 yo) at a large adult burn center have a mortality rate of near 70% despite average burn sizes of near 15% total body surface area (TBSA) [4,5]. Infection was a common complication in that population. Thus, any size of burn that is complicated by infection is a major concern and can lead to multi-organ failure and death.

Burn injuries have been reported to alter adaptive immune functions, which may further increase susceptibility to infection. Valvis *et al* reported that cutaneous burn injuries induce increased numbers of T cells in inguinal lymph nodes but cause suppressed splenic T cell cytokine production [6]. Dendritic cell dysfunction after burns may also contribute to impaired T cell functions [7]. In addition to the immune dysfunction caused by burn trauma, sepsis also induces immunosuppression due, in part, to defects in adaptive immune system activity [8,9]. Thus, the presence of sepsis may further compromise the ability of the burned host to eradicate primary infections and increase susceptibility to secondary nosocomial infections. Furthermore, sepsis induced multi-organ injury significantly contributes to increased morbidity and mortality [10–12]. When combined with infection, the physiologic disturbances caused by burn injury often lead to multiorgan failure, and death [13–16]. In fact, infection is the most common cause of death in burn victims that survive the initial burn trauma and is a major cause of prolonged hospitalization. The problem of infection in burn victims is becoming increasingly troublesome due to the emergence of antibiotic resistant bacteria such as *Pseundomonas sp* and *Acinitobacter sp* as common pathogens in this population [17,18]. Consequently, there is great interest in developing strategies to decrease the incidence and severity of infections in burned patients. Immunotherapies aimed at strengthening host resistance to infection are one approach that could be efficacious in this setting [8,19].

Members of the interleukin (IL)-2 receptor activating family of cytokines, such as IL-7 and IL-15, have been shown to improve survival in an experimental model of polymicrobial sepsis caused by cecal ligation and puncture (CLP) [20,21]. In addition to its role in treating bacterial infections, IL-15 has been extensively studied for its protective anti-tumor efficacy in several cancer pre-clinical studies [22]; as well as its ability to augment the efficacy of HIV vaccines [23]. The major immune cells that produce IL-15 include dendritic cells, macrophages, monocytes, endothelial cells, stromal cells and renal epithelial cells, which transpresent IL-15 in association with the IL-15 receptor alpha chain [24–27]. IL-15 can be induced by various stimuli including endotoxin, interferons α/β/γ, double stranded RNA [28], and infection with viruses [29]. Transpresented IL-15 signals its action through a heterodimeric receptor that shares the IL-2R/IL-15Rβ (CD122) beta and common gamma chains [30]. Functionally, IL-15 has been characterized as a T cell growth factor and stimulates T cell proliferation (memory CD8^+^ T cells preferentially), immunoglobulin synthesis by B cells and is essential for the growth and survival of natural killer (NK) and NKT cells [31]. Mice lacking IL-15 or IL-15 receptor-α (IL-15Rα) have deficiency of these target cells in their immune system [32,33]. IL-15 is also known to positively impact the functioning of innate immune cells, including protection of neutrophils from apoptosis and modulation of neutrophil phagocytic functions [34]; acting as an inhibitor of apoptosis and serving as a growth factor for mast cells [35]; increasing phagocytic action and cytokine secretion of macrophages [36]; and inducing maturation and inhibition of apoptosis among dendritic cells [37]. Therefore, IL-15 is an essential cytokine to sustain the normal coordinated functioning of both the innate and adaptive immune systems.

As noted above, IL-15 producing cells transport IL-15 to their surface complexed with IL-15 receptor-alpha (IL-15Rα) and present it to target cells (memory CD8+ T, NK and NKT cells) expressing IL-15 receptor β and common γ chains, through a unique mechanism called as trans-presentation [38–40]. Rubinstein and colleagues have shown that combination of IL-15 and IL-15Rα in solution generates a complex, termed as IL-15 superagonist (IL-15 SA), that possesses a significantly enhanced half-life compared to native IL-15 and is more potent *in vivo* [41]. Treatment with IL-15 SA has been shown to prevent T cell apoptosis, ameliorate innate and adaptive immune system dysfunction and reduce mortality in the CLP model of sepsis [20]. Furthermore, treatment with native IL-15 has been shown to protect against *Escherichia coli*-induced septic shock [42]; enhance CD8^+^ T cell-mediated killing of pathogens such as *Cryptococcus neoformans* [43]; improve clearance of *Salmonella* [44]; and improve survival in a murine model of *Mycobacterium tuberculosis* [45]. These studies indeed suggest that treatment with IL-15 or IL-15 SA might be helpful to prevent and/or treat the burn injury-associated infections and sepsis.

Despite reports of altered adaptive immune function in burned mice, previous studies have not investigated those alterations in detail. Furthermore, there are no reports on the effect of combined burn injury and infection on adaptive immune cell populations. In the present study, we investigated the effect of combined cutaneous burn and infection on adaptive immune function. We further assessed the impact of IL-15 SA treatment on expansion of T, NK and NKT cells and its ability to protect against *Pseudomonas aeruginosa-*induced sepsis in a mouse model of cutaneous burn injury.

## Materials and Methods

### Mouse Model of Burn Injury

All animal procedures were in accordance with the National Institutes of Health guidelines and were approved by the Institutional Animal Care and Use Committee at Vanderbilt University Medical Center. Eight to ten week-old male BALB/c mice were purchased from Harlan Laboratories (Indianapolis, IN). A well-established mouse model of full-thickness cutaneous burn injury was used [46–49]. Briefly, mice were housed in an appropriate institutional care facility and allowed to acclimate for 1 week after arrival. Buprenorphine (0.1 mg/kg, subcutaneously) was administered 30 minutes prior to burn injury for analgesia. Under general anesthesia using 2.5% isoflurane, the dorsal surface of mice was shaved and 1 mL normal saline was injected subcutaneously into the burn target area to prevent injury to the underlying tissues. Using a molded template containing a rectangular opening, an approximately 35% total body surface area scald burn was induced by exposing the shaved dorsal area to 97–98°C water for 10 seconds. Immediately afterwards, 2 mL Lactated Ringers solution was administered by the intraperitoneal route for fluid resuscitation. All mice received a second injection of buprenorphine at 8−12 hours after the burn procedure. Additional buprenorphine was administered if needed (as indicated by decreased movement, eating, or drinking, restlessness or abnormal posture) twice daily after burn injury. The burn injured mice were housed individually in sterile cages and were provided sterile water and food. Sham mice underwent the same experimental procedure except that mice were exposed to room temperature water.

### Burn Wound Infection

**Fig 1:**
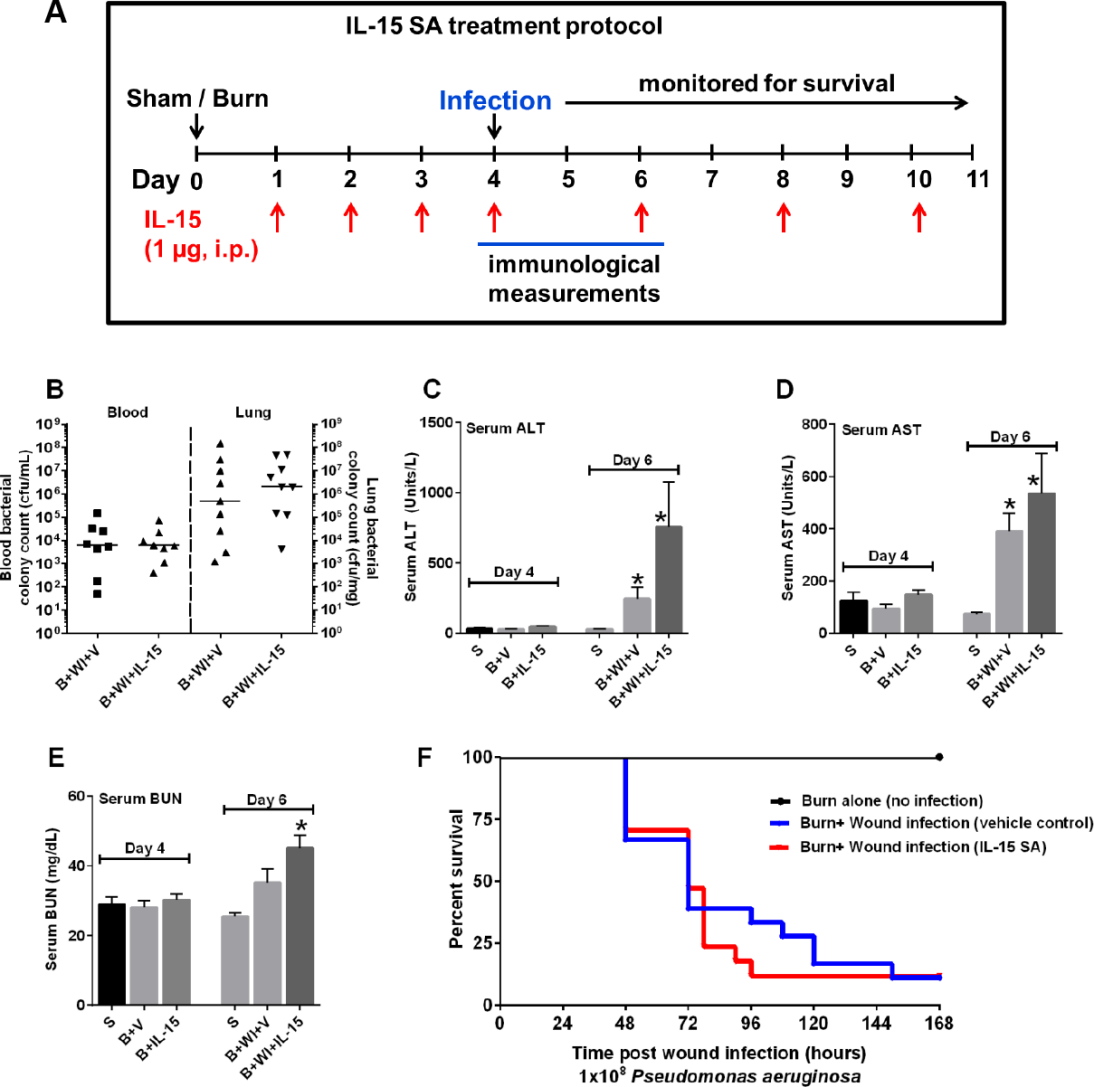
IL-15 SA treatment does not protect against burn wound infection induced organ injury and decreased survival. (Among the respective groups, the X axis symbols mean the following: S = Sham (no burn), B = burn injury, V = Vehicle treatment, WI = Wound infection and IL-15 SA = interleukin 15 super agonist treatment). IL-15 SA was administered at a dose of 1 μg via i.p route starting a day after burn injury. (A) Time line depicting the burn injury and wound infection model along with IL-15 SA treatment. **(B)** To evaluate the systemic spread of infection, *Pseudomonas aeruginosa* colony counts were measured on day 2 post wound infection and the graph depicts colony forming units of the bacteria in the blood (left half) and the lungs (right half). Following parameters were measured on day 4 and day 6 (equivalent to day 2 post wound infection) post burn injury and wound infection **(C)** serum alanine aminotransferase, ALT; **(D)** serum aspartate amino transferase, AST; and **(E)** serum blood urea nitrogen. n = 8–10 in each group and P<0.05. **(F)** Survival study: Three groups of mice including—burn injured (n = 18, black line); burn injured and vehicle treated wound infected mice (n = 18, red line); and burn injured and IL15 SA treated wound infected mice (n = 18, blue line) were monitored for survival for seven days post *Pseudomonas aeruginosa* wound inoculation. The graph represents results from three separate experiments. *significantly different as compared to respective day 4 and day 6 sham groups. For each of the figures, x-axis labels indicate the following: **S** = Sham (no burn injury); **B+V** = burn injury + vehicle treated mice for 4 days; **B+IL-15 SA** = burn injury + IL-15 SA treated mice for 4 days; **B+WI+V** = burn injury + wound infection performed on day 4 + vehicle treated mice for 6 days; **B+WI+IL-15** = burn injury + wound infection performed on day 4 + IL-15 SA treated mice for 6 days.

The burn wound was inoculated on the surface with *Pseudomonas aeruginosa* bacteria obtained from American Type Culture and Collection (Manassas, VA; ATCC 19660). The culture was grown in tryptic soy broth and diluted in sterile saline solution prior to inoculation. On day 4 post burn, the burn wound was inoculated on the surface with 1 x 10^8^ colony forming units (cfu) of *Pseudomonas aeruginosa* to induce wound infection. Fig 1A provides a graphical representation of the burn injury and wound infection time line. In some experiments, burned mice were inoculated by intraperitoneal injection with 1 x 10^8^ cfu of *Pseudomonas* on day 4 post-burn. That model induces an acute infection and allows for reproducible assessment of bacterial clearance and leukocyte recruitment into the site of infection.

### IL-15 Superagonist (IL-15 SA) Preparation

Mouse IL-15 Receptor α subunit Fc chimera (IL-15Rα) was obtained from R&D systems (Minneapolis, MN, Cat. No. 551-MR-100). Recombinant IL-15 was purchased from eBiosciences (San Diego, CA, Cat no. 34-8151-85). IL-15 superagonist was prepared as described previously [20,41]. Briefly, 20 μg of IL-15 and 90 μg of IL-15Rα were incubated in 400 μL of sterile phosphate buffered saline (PBS) at 37°C for 20 min to form the IL-15/IL-15Rα complex (IL-15 SA). That preparation was further diluted with sterile PBS to prepare a stock concentration of 2 μg IL-15 SA and stored at -80°C for future use.

### IL-15 Treatment Protocol

The IL-15 SA doses reported in this study are based on the amount of IL-15 present in the IL-15 SA complex. A study by Inoue *et al*., demonstrated IL-15 SA-mediated protection in a CLP model of sepsis using a dose of 1.5 μg IL-15 SA. Recently published study from our laboratory shows that repeated systemic administration of 2 μg IL-15 SA leads to significant toxicity in the mice [50], with no toxicity observed at doses of 1 μg or less. Therefore, we utilized a dose of 1 μg IL-15 SA (in 0.5mL PBS) administered via intraperitoneal injection. IL-15 SA was administered daily starting one day post burn injury until day 4 and then every other day thereafter, as shown below. Equivalent volume (0.5mL) of PBS was administered to the vehicle treated groups. Blood, spleen and liver were harvested on day 4 post burn (prior to infection and after three doses of IL-15 SA) and on day 2 following wound infection to assess lymphocytes numbers and activation. Mice were monitored for 7 days post wound infection in survival studies. Fig 1A provides graphical representation of the IL-15 SA treatment time line.

### Preparation of Single Cell Suspensions

#### Peritoneal leukocyte harvesting

Intraperitoneal leukocytes were obtained by lavage of the peritoneal cavity with 2 mL of cold PBS. Cells were counted, centrifuged (300 x g for 10 minutes at 4°C) and resuspended in PBS at desired cell concentrations. Cell counting was performed using a TC20 automated cell counter (Bio-Rad, Hercules, CA).

#### Splenocyte preparation

Single cell suspensions of splenocytes were prepared by gently pressing the isolated spleen through 70 μm cell strainer. The splenocytes were centrifuged (300 x g for 10 minutes at 4°C) and red blood cells in the splenocyte pellet were lysed with Red Blood Cell Lysis Buffer (Sigma Life Sciences, St Louis, MO). The cell count per spleen was then measured using a TC20 cell counter; splenocytes were centrifuged (300 x g for 10 minutes at 4°C) and resuspended in PBS to achieve a concentration of 1x10^7^ cells/mL, for further analysis using flow cytometry.

#### Liver leukocyte preparation

As described previously [50], liver was perfused with cold PBS through injection into the left ventricle of the heart and then harvested. Liver tissue was gently pressed through a 70 μm cell strainer. The resulting homogenate was resuspended in 10 mL of 37.5% Percoll Plus (GE Healthcare Life Sciences) and centrifuged (680 x g for 12 minutes at room temperature). The supernatant containing the hepatocytes was discarded and red blood cells lysed. The remaining mononuclear immune cells were counted and resuspended in PBS to achieve a concentration of 1 x 10^7^ cells/mL, for flow cytometry analysis.

### Flow Cytometry

Leukocytes isolated from peritoneal cavity, spleen and liver tissues were suspended in cold PBS (1 x 10^7^ cells/mL) and incubated with 1 μl/mL anti-mouse CD16/32 (eBioscience, San Diego, CA) for 5 minutes to block non-specific Fc receptor-mediated antibody binding. One million cells were then transferred to polystyrene tubes and incubated with 0.5 μg of fluorochrome-conjugated specific antibodies or isotype control antibodies (4°C, 30 minutes), followed by washing with 2 mL cold PBS and centrifugation (300 x g for 5 minutes). The cell pellet was then resuspended in 250 μL cold PBS. Flow cytometry was performed using BD Accuri C6 instrument (BD Biosciences, San Diego, CA). Data were analyzed using Accuri C6 software. The following fluorochrome conjugated anti-mouse antibodies (eBioscience, San Diego, CA) were used: anti-CD3-FITC, anti-CD4-PerCPCy5.5, anti-CD4-FITC, anti-CD8-PE, anti-CD19-PE, anti-F4/80-FITC, anti-CD49b-APC, anti-CD69-PE, anti-CD69-PerCPCy5.5 and respective isotype controls.

### Measurement of Bacterial Counts and Temperature

Serial dilutions of blood, peritoneal fluid or lung tissue homogenates were grown on tryptic soy agar overnight to determine colony forming units (CFU) of *Pseudomonas aeruginosa* per ml of blood or per gram of tissue. Bacterial counts were measured on day 2 post wound infection and 6 hours post intraperitoneal inoculation of *Pseudomonas aeruginosa*. Body temperature was measured using a lubricated rectal temperature probe and digital thermometer (Physitemp Instruments INC., Clifton, NJ, USA).

### Measurement of Complete Blood Count (CBC) and Organ Injury Markers

Whole blood was harvested by carotid artery laceration under general isoflurane anesthesia. Blood was collected in K3EDTA tubes (Greiner Bio-Obe, Kremsmunster, Austria) and kept on ice until CBC and differential leukocyte counts analysis. CBC measurements were performed using a Forcyte veterinary hematology analyzer (Oxford Science, Oxford, CT). Remaining blood was centrifuged (4750 rpm for 15 minutes at 4°C) to collect plasma for cytokine analyses or measurement of liver injury markers–alanine aminotransferase (ALT) and aspartate amino transferase (AST); and kidney injury marker—blood urea nitrogen (BUN). These were measured using Vet Axcel Chemistry Analyzer in the Translational Pathology Shared Resource facility at Vanderbilt University.

### Cytokine Measurements

Concentrations of IL-6, IL-10, TNF-α, IFN-γ, KC and MIP-2 were measured in plasma using a Bio-Plex Multiplex Assay and read with the Bio-Plex Magpix Multiplex Reader (Bio-Rad, Hercules, CA). Results were analyzed using Bio-Plex Manager Software 6.1.

### Survival Study

Following wound infection with *Pseudomonas aeruginosa*, mice were monitored at least three times daily for seven days. Additionally, the animal care veterinary technicians also monitored the health of mice on a daily basis. Mice were checked for signs of morbidity including decreased activity and response to stimuli combined with abnormal posturing and labored breathing. Core body temperature was also measured using a lubricated rectal temperature probe and digital thermometer (Physitemp Instruments INC., Clifton, NJ, USA), as an indicator of morbidity [10,50]. Mice displaying signs of morbidity and/or body temperature below 30°C, were humanely euthanized under isoflurane anesthesia as non-survivors. To assure proper euthanasia, the unconscious mice underwent cervical dislocation and mortality was confirmed.

### Statistical Analysis

Graph preparations and data analyses were performed using Prism 6.0 (GraphPad Software Inc., San Diego, CA). Data with three or more groups were analyzed using a one-way ANOVA followed by the Tukey multi-comparison post-hoc test. Comparison between two groups was analyzed using Students T test. All data values are presented as mean ± SEM, except for bacterial counts, for which median values are designated. The median values derived from bacterial clearance studies were evaluated using the non-parametric Mann Whitney test. P-values less than 5% levels were considered statistically significant. Survival curves were analyzed using a Mantel-Cox log rank test.

## Results

### Burn Wound Infection Significantly Decreases Blood Lymphocyte Counts and Induces Organ Injury and Sepsis, which Is Not Improved by IL-15 SA Treatment

Wound infection was induced by topical application of *Pseudomonas aeruginosa* on day 4 after burn injury (Fig 1A). This time point of inoculation represents a clinical scenario wherein patients typically develop infection after burn injury [51]. Upon inoculation of the wound, the bacteria gradually colonize the injured area and spreads systemically into surrounding tissues, blood and eventually to distant organs, namely the lungs [49]. As shown in Fig 1B, *Pseudomonas* was present in the blood and lungs on day 2 after wound infection, demonstrating systemic spread. However, no difference in blood and lung bacterial counts was observed when comparing vehicle- and IL-15 SA-treated mice (Fig 1B). Burn wound sepsis also led to a significant increase in serum ALT and AST concentrations, as compared to sham or burn only mice, implying liver injury (Fig 1C and 1D). Although a trend towards higher ALT and AST concentrations was observed in infected mice treated with IL-15 SA, differences were not statistically significant among groups. Serum BUN concentrations were significantly higher in infected mice treated with IL-15 SA compared to sham and vehicle-treated mice, suggesting kidney injury in that group (Fig 1E). These findings were associated with approximately 80% mortality on day 4 after wound infection in both vehicle- and IL-15 SA-treated mice, with no significant difference noted among groups (Fig 1F).

**Fig 2:**
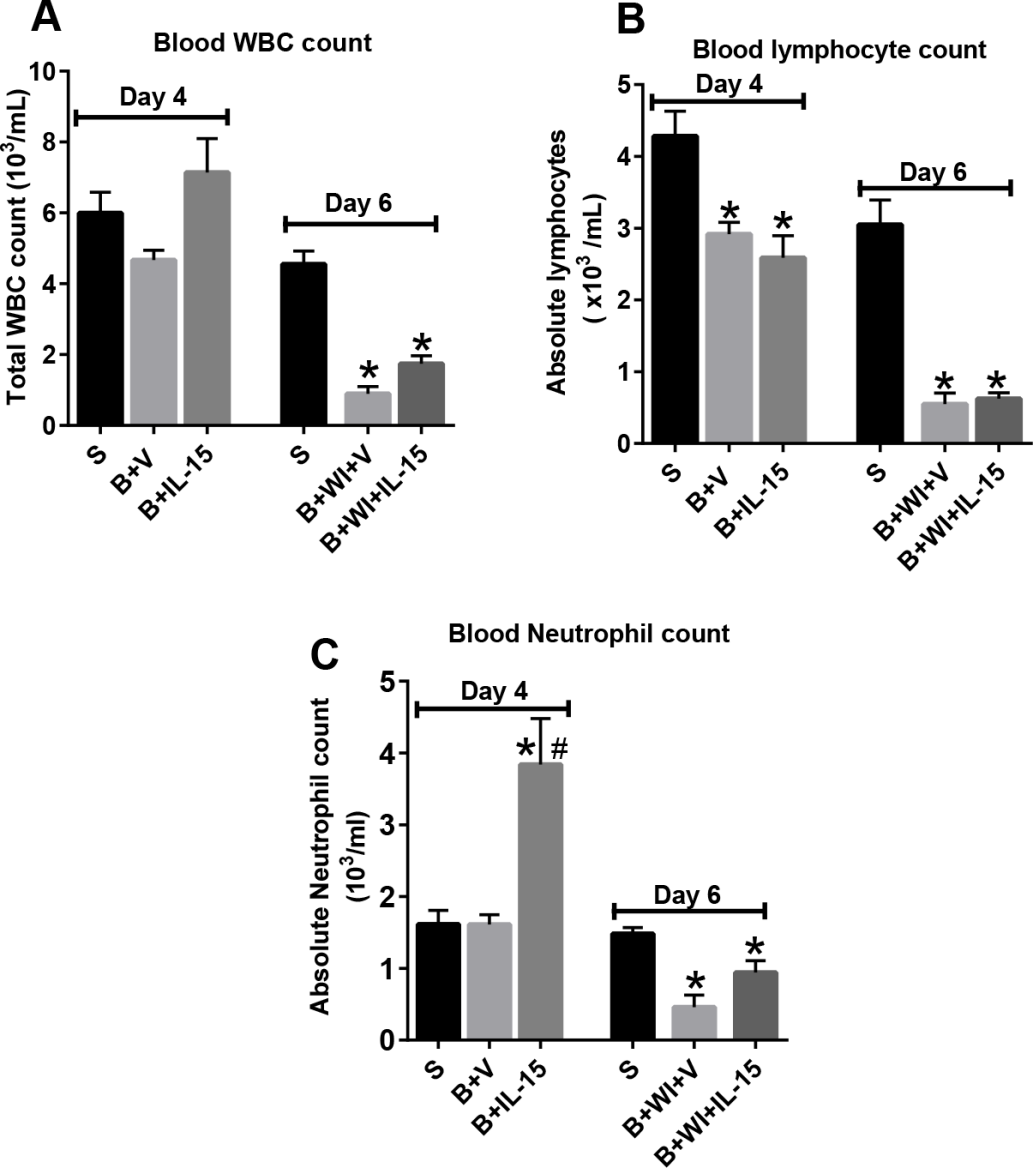
Burn wound infection caused decline in white blood cell and lymphocyte counts. **(A)** Total white blood cell counts. **(B)** Blood lymphocyte counts. (C) Blood neutrophil counts. n = 8–10 in each group, *significantly different as compared to respective day 4 and day 6 sham groups; #significantly different from burn plus vehicle group (B+V) and P<0.05.

Burn injury alone induced a significant decrease in blood absolute lymphocyte count, but not total white blood cell count, on day 4 post-burn in vehicle- and IL-15 SA-treated mice (Fig 2A and 2B). Both total white blood cell and absolute lymphocyte counts were significantly (>80%) decreased at 2 days after burn wound infection in both groups (Fig 2A and 2B, day 6 post-burn) compared to sham controls and burned mice on day 4. On the other hand, burn injury alone did not cause any change in blood neutrophil counts, but IL-15 SA treatment significantly increased it, at day 4 post burn (Fig 2C). Wound infection lead to a significant decline in the blood neutrophil counts both in the vehicle- and IL-15 SA-treated groups at day 2 post infection (Fig 2C).

### IL-15 SA Treatment Significantly Increases CD4^+^, CD8^+^ and B Lymphocyte Counts in the Spleen and Liver after Burn Injury, while Subsequent Wound Infection Decreases These Cells

**Fig 3:**
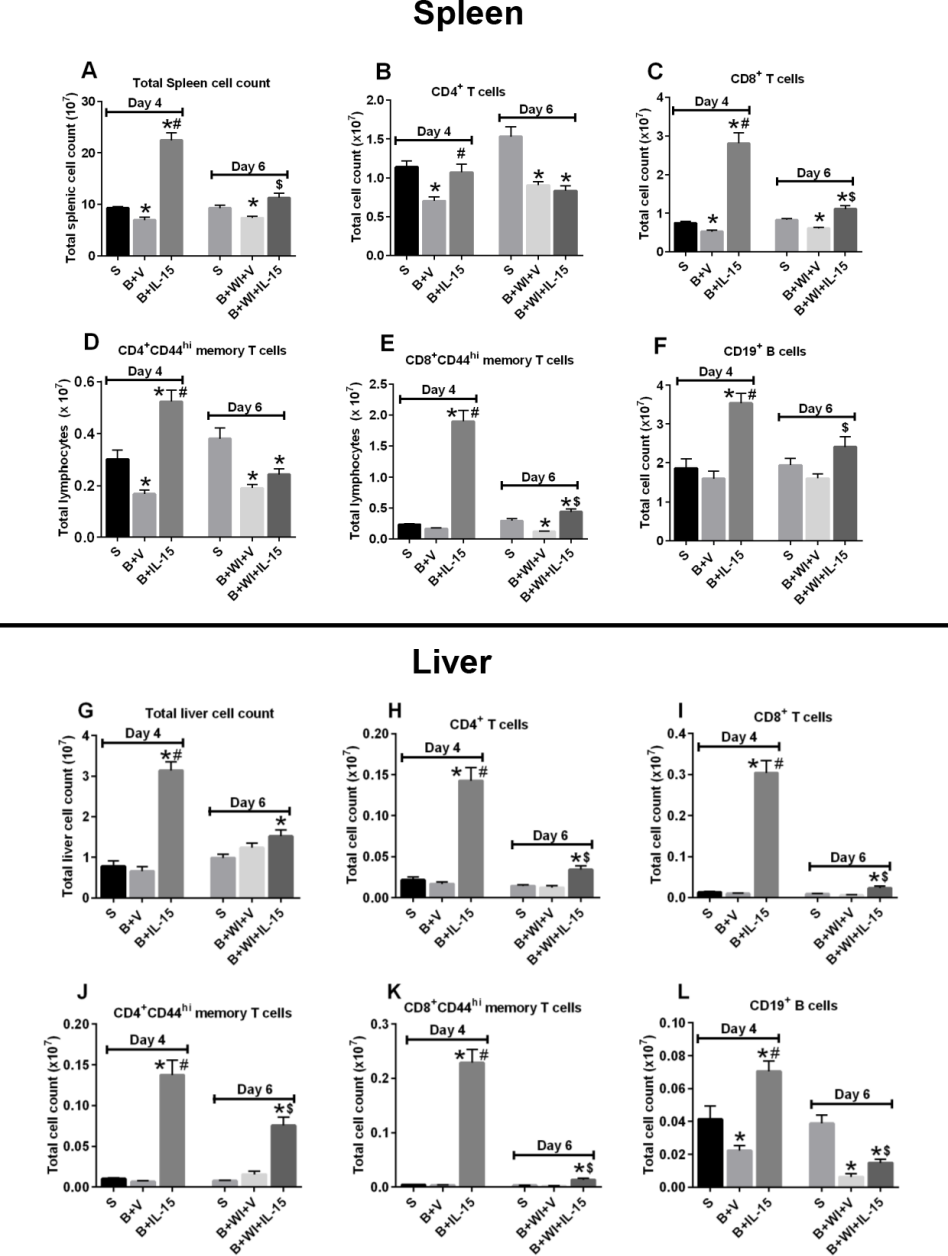
Effect of burn injury and wound infection with or without IL-15 SA treatment on CD4^+^, CD8^+^ T and CD19^+^ B lymphocyte cell counts. Wound infection and sepsis was induced by inoculation of *Pseudomonas aeruginosa* on day 4 post burn injury. Upper half of the panel depicts cell counts in the spleen and lower half of the panel depicts cell counts in the liver. Spleen and liver cell counts were analyzed on day 4, and day 6 (equivalent to day 2 post wound infection) post burn injury. **(A** and **G)** Total immune cell counts among the respective groups of the single cell suspensions prepared from the spleen and liver. Flow cytometry was used to measure the individual T and B cell lymphocyte cell population in the spleen and liver. The bar graph represents the total cell counts of the CD4^+^ T cells **(B** and **H)**, CD4^+^ CD44^hi^ memory T cells **(D** and **J**), CD8^+^ T cells **(C** and **I**), CD8^+^ CD44^hi^ T cells **(E** and **K)**, and CD3^-^CD19^+^ B cells **(F** and **L**); in the spleen and liver. n = 8–12 in each group and P<0.05. *significantly different as compared to respective day 4 and day 6 sham groups; #significantly different from day 4 burn injury group (B+V) and $-significantly different from day 6 burn injury + vehicle treated wound infected group (B+WI+V).

As shown in Fig 3A–3F, burn injury significantly decreased total splenocyte, CD4^+^ T, CD8^+^ T and memory CD4^+^CD44^+^ T cell counts but not memory CD8^+^CD44^+^ T or B cell counts in spleen. With the exception of a decrease in B cell numbers, burn injury did not decrease any of those cell populations in liver (Fig 3G–3L). IL-15 SA treatment (1 μg, i.p, for 3 days starting a day after burn injury) significantly increased the numbers of total leukocytes, T cells and B cells in spleen and liver at day 4 after burn (Fig 3A–3L). However, on day 2 post *Pseudomonas aeruginosa* wound inoculation, infection led to a significant loss of the CD4^+^, mCD4^+^, CD8^+^, mCD8^+^ cells in the spleen as compared to the sham mice (day 6) (Fig 3A–3E). None of the T cell counts were decreased in the liver post infection in vehicle treated group as compared to sham mice (day 6) (Fig 3G–3K). Infection alone (vehicle treated) did not affect CD19^+^ B cell numbers in the spleen (Fig 3F), but significantly decreased it in liver (Fig 3L). Although IL-15 SA-treated mice had relatively higher cell counts after wound infection as compared to the respective (day 6) vehicle treated infected mice (Fig 3), infection still caused a significant decline in all the T cell subsets and B cell numbers as compared to IL-15 SA expanded day 4 cell numbers (Fig 3).

### IL-15 SA Induces T and B Lymphocytes Activation

**Fig 4:**
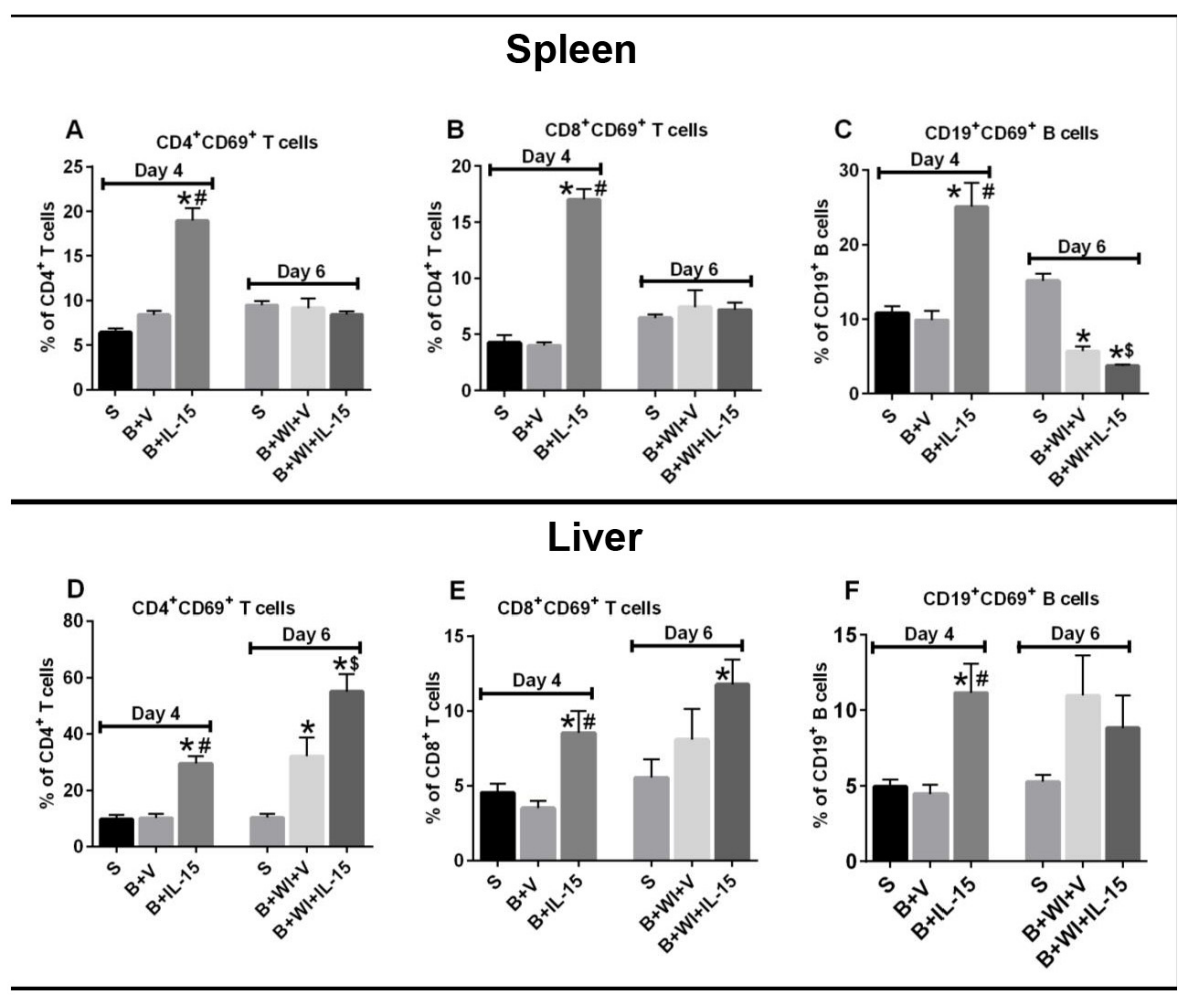
Effect of burn injury and wound infection with or without IL-15 SA treatment on CD4^+^, CD8^+^ T and CD19^+^ B lymphocytes activation status. CD69 surface expression was used as a marker of cellular activation and measured by flow cytometry. Upper half of the panel depicts data for spleen cells and lower half of the panel depicts data for liver tissue. Percentage of CD69 expression was measured on CD4^+^ T cells **(A** and **D)**, CD8^+^ T cells **(B** and **E**), and CD3^-^CD19^+^ B cells **(C** and **F**); in the spleen and liver. n = 8–12 in each group and P<0.05. *significantly different as compared to respective day 4 and day 6 sham groups; #significantly different from day 4 burn injury group (B+V), and $—significantly different from day 6 burn injury + wound infection + vehicle treated group (B+WI+V).

IL-15 SA treatment significantly activated CD4^+^ T, CD8^+^ T and CD3^-^CD19^+^ B lymphocytes in the spleen and liver, as measured by increased percentage of cells expressing the early activation marker CD69 (Fig 4). IL-15 SA-induced cellular activation declined among splenic CD4^+^ and CD8^+^ T cells after wound infection as compared to day 4 post burn injury and were comparable to the respective day 6 sham group mice (Fig 4A and 4B). On the contrary, wound infection led to an increase in the activation of CD4^+^ and CD8^+^ T cells in the liver as compared to the respective sham control mice. (Fig 4D and 4E). IL-15 SA treatment accentuated the CD4^+^ T cell activation more so than the CD8^+^ T cell activation in the liver after infection. In this regards, IL-15 SA caused a substantially higher activation of CD4+ T cells (~ 55% CD69 expression) post infection as compared to the sham (~10%) and vehicle treated burn wound infected group (~32%). On the other hand, IL-15 SA treatment caused CD8+ T cell activation (~12% CD69 expression) post infection, which was significantly higher as compared to sham (5%) but not statistically different as compared to the vehicle treated burn wound infected group (~8%) (Fig 4D and 4E). IL-15SA treatment also activated B cells in spleen and liver from burned mice (Fig 4C and 4F). Burn wound infection decreased CD19^+^ B cell activation in the spleens of vehicle and IL-15SA-treated mice. However, B cell activation was sustained in the liver after burn wound infection (Fig 4C and 4F).

### IL-15 SA Treatment Significantly Expanded NK and NKT Cells and Activated NK Cells in the Spleen and Liver

**Fig 5:**
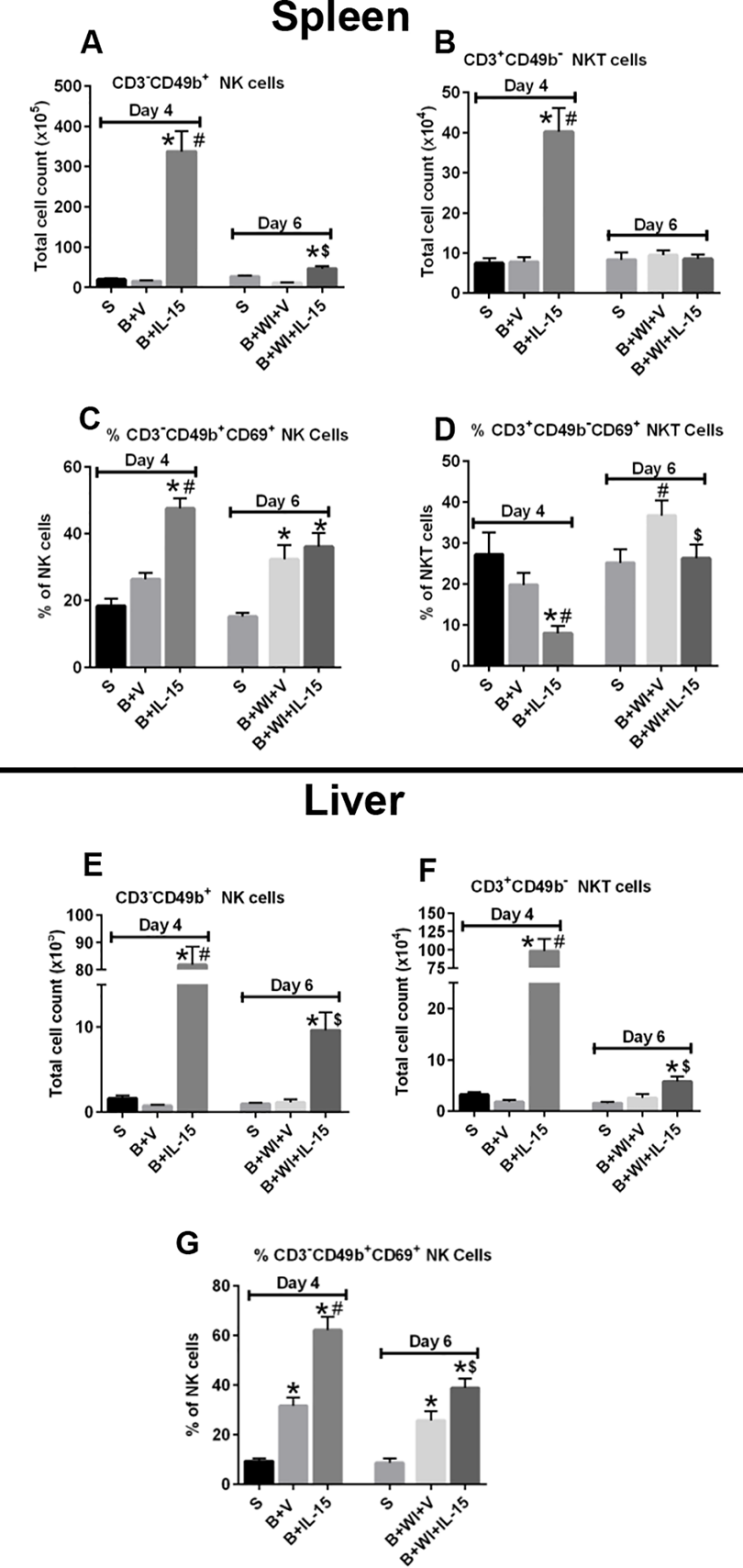
IL-15 SA treatment elicits expansion of NK and NKT cells post burn injury and wound infection causes their decline. Upper half and lower half of the panel depicts cell counts and activation status (CD69^+^) of NK (CD3^-^CD49b^+^) and NKT (CD3^+^CD49b^-^) cells in the spleen and liver respectively. The graphs **A** and **E** depict NK cell counts in the spleen and liver respectively; and the graphs **B** and **F** show the NKT cell counts in the spleen and liver respectively. The graphs **C** and **G** depict NK cell CD69 expression in the spleen and liver respectively; and the graph **D** shows the NKT cell CD69 expression in the spleen. n = 8–10 in each group and P<0.05. *significantly different as compared to respective day 4 and day 6 sham groups; #significantly different from day 4 burn injury group (B+V), and $—significantly different from day 6 burn injury + wound infection + vehicle treated group (B+WI+V).

Consistent with earlier reports, IL-15 SA treatment significantly increased NK (CD3^-^CD49b^+^) and NKT (CD3^+^CD49b^+^) cell numbers both in the spleen and liver on day 4 after burn injury (Fig 5A, 5B, 5E and 5F) as compared to the sham and vehicle treated mice. However, wound infection led to a significant decline in the expanded NK and NKT cell numbers, both in the spleen and liver. Burn injury alone significantly increased the activation (increased CD69 expression) of NK cells in the liver, but not spleen, on day 4 in the vehicle treated group. IL-15 SA treatment induced activation of NK cells in the spleen and liver of burned mice (Fig 5C and 5G). On the contrary, IL-15 SA treatment significantly attenuated the basal NKT cell activation after burn injury alone and there was a trend towards increase in the basal NKT cell activation after wound infection in the spleen (Fig 5D).

### Wound Infection Increased Pro-Inflammatory Cytokine Production, which Was Not Affected by IL-15SA Treatment

**Fig 6:**
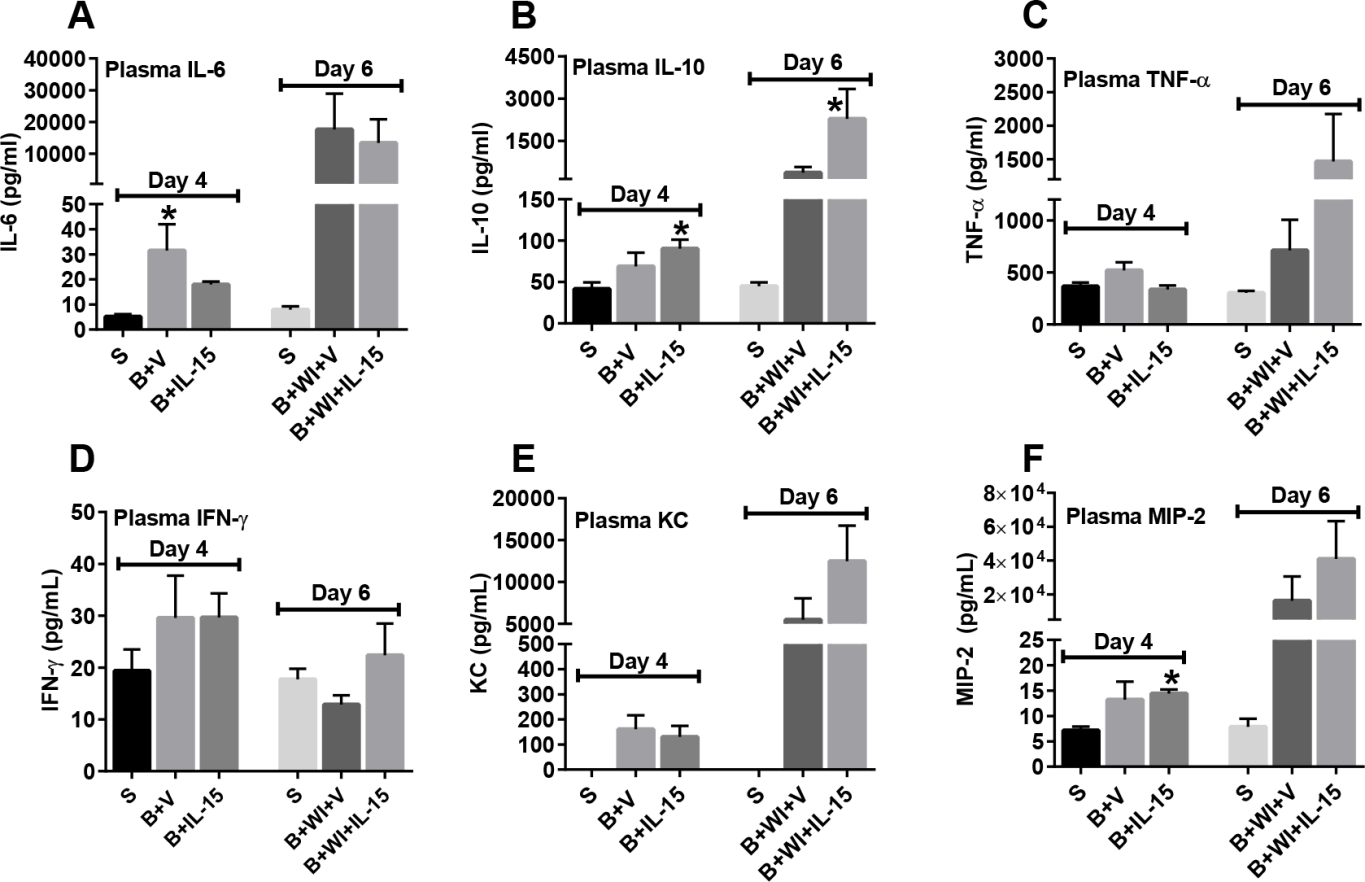
Plasma cytokine levels post burn injury and wound infection. Plasma cytokines including (A) IL-6, (B) IL-10, (C) TNF-α, (D) IFN-γ, (E) KC and (F) MIP-2 were measured using Bio-Rad Bio-Plex assay. n = 6–8 in each group and P<0.05. *significantly different as compared to respective day 4 and day 6 sham groups.

As shown in Fig 6, burn wound infection substantially increased plasma concentrations of the cytokines IL-6, IL-10, TNF-α, KC and MIP-2. Treatment with IL-15 SA did not modify post-infection cytokine production when compared to vehicle-treated mice.

### IL-15 SA Treatment Inhibits Neutrophil Recruitment to the Site of Local Infection and Fails to Enhance Bacterial Clearance

**Fig 7:**
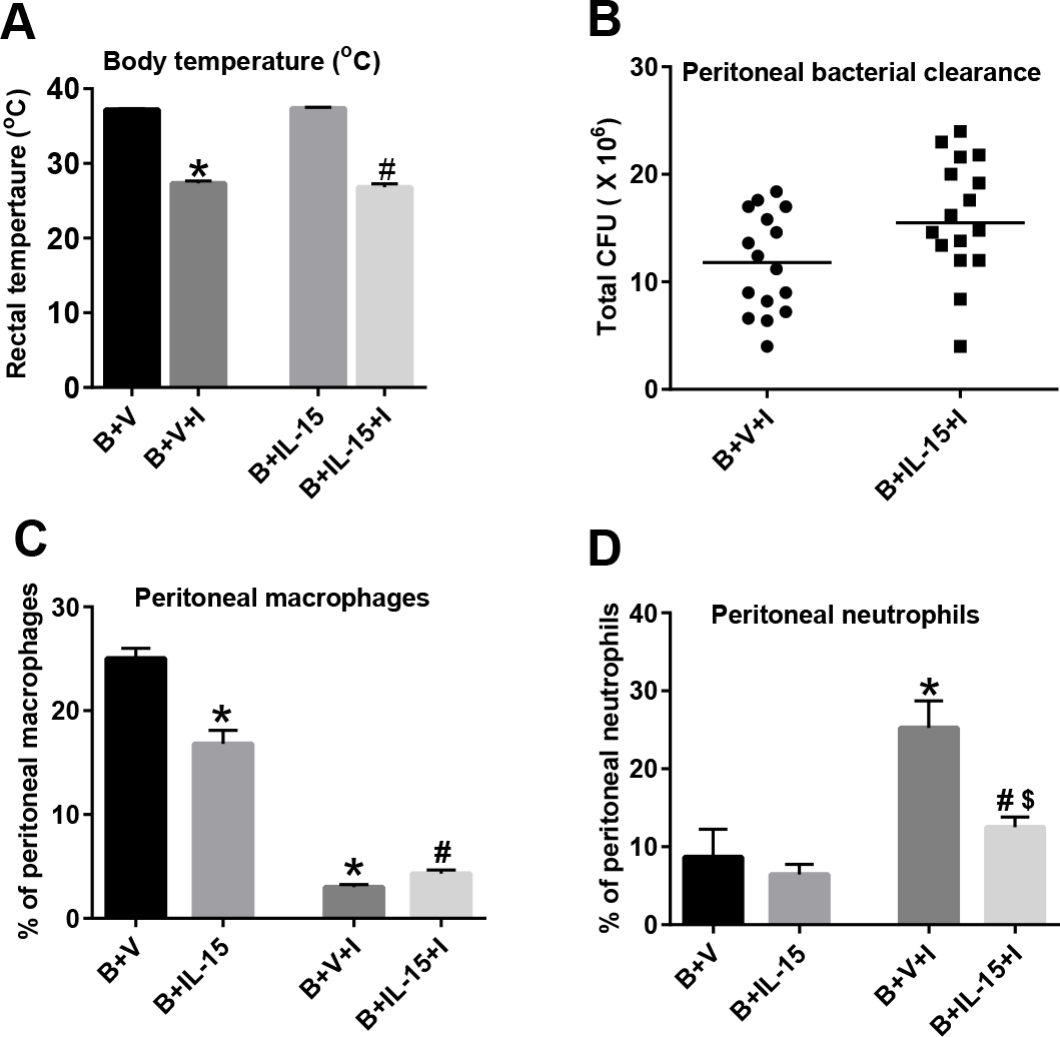
Effect of IL-15 SA treatment on neutrophil recruitment and bacterial clearance upon intraperitoneal infection. *Pseudomonas aeruginosa* (1x10^8^ CFU) was inoculated in the peritoneum of the mice via i.p injection. This model was used to assess if IL-15 SA treatment causes neutrophil recruitment and improves bacterial clearance. Mice were divided into four groups: 1) **B+V** = burn + vehicle treated non-infected mice; 2) **B+IL-15** = burn + IL-15 SA treated non-infected mice; 3) **B+V+I** = burn + vehicle treated infected mice; 4) **B+IL-15+I** = burn + IL-15 SA treated infected mice. **(A)** At 6 hours post i.p infection, core body temperature was measured using a rectal temperature probe. Also, peritoneal lavage was performed using 2 mL sterile PBS solution and used for bacterial clearance **(B)**; macrophage **(C)** and neutrophil **(D)** cell counts measurements using flow cytometry. n = 8–16 in each group and P<0.05. *significantly different as compared to B+V group; #significantly different from B+IL-15 group, significantly different as compared to B+V group $—significantly different from B+V+I group.

In further studies, we utilized a two-hit model of infection, in which burn injured mice received intraperitoneal challenge with *Pseudomonas aeruginosa* on day 4 after burn injury to assess leukocyte recruitment to the site of infection. The effect of IL-15 SA treatment upon neutrophil recruitment and bacterial burden was assessed in the peritoneal fluid at 6 hours after bacterial inoculation. Body temperature was measured as an index of infection severity (Fig 7A). Infection significantly lowered the core body temperature of mice, both with and without IL-15 SA treatment. IL-15 SA treatment did not change the bacterial burden in the peritoneal cavity as compared to the vehicle treated mice (Fig 7B). Furthermore, IL-15 SA treatment significantly reduced the percentage of peritoneal macrophages (F480^+^Ly6G^-^) after burn injury (Fig 7C). Infection markedly decreased intraperitoneal macrophages in both vehicle and IL-15SA-treated mice. Infection increased the percentage of intraperitoneal neutrophils (Ly6G^+^F4/80^-^) in vehicle and IL-15SA-treated mice, but IL-15 SA treatment attenuated neutrophil recruitment at the site of bacterial inoculation (Fig 7D).

### Intraperitoneal Infection Significantly Increased Cytokine Production, which Was Amplified by IL-15SA Treatment

**Fig 8:**
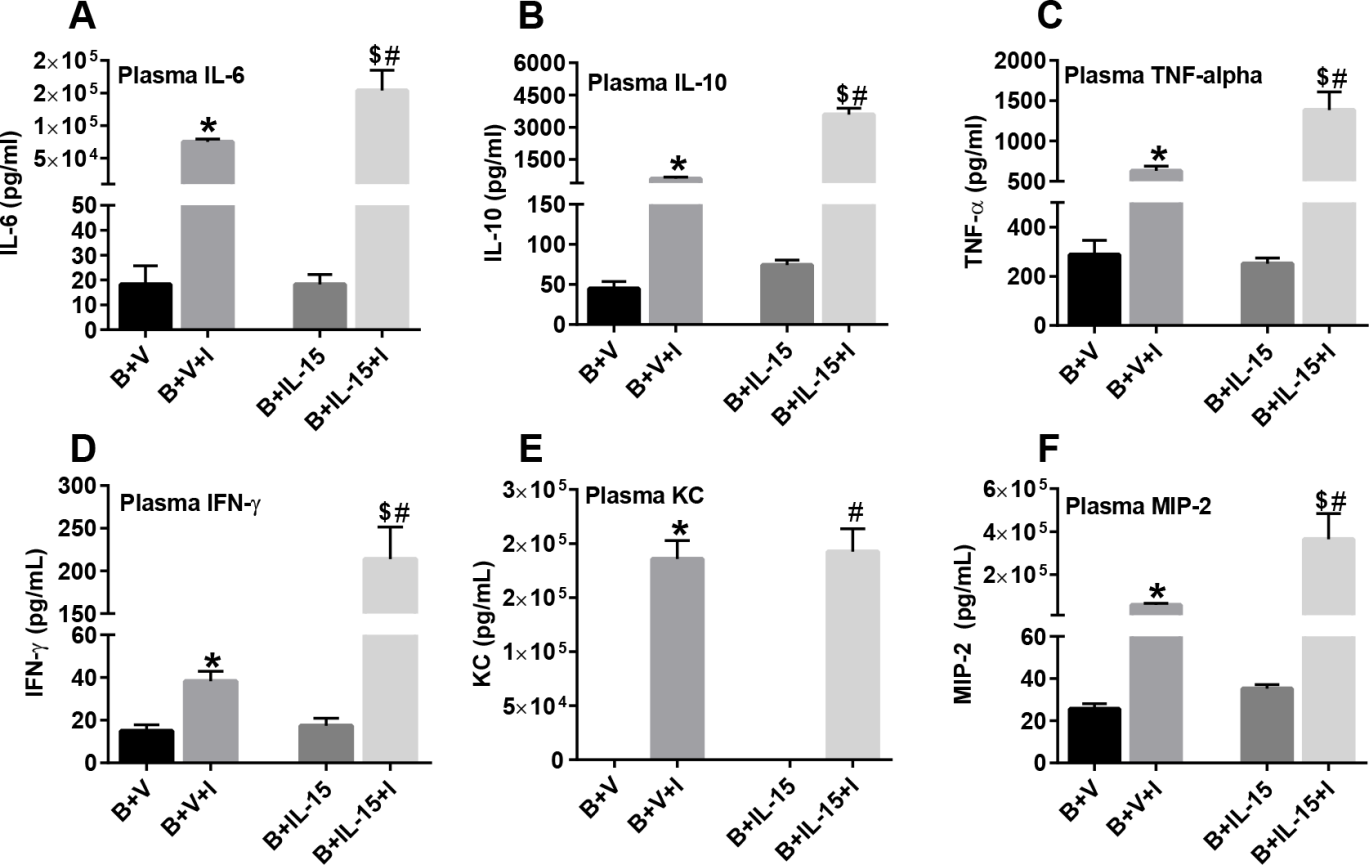
Plasma cytokine levels post intraperitoneal infection with *Pseudomonas aeruginosa.* Plasma cytokines including (A) IL-6, (B) IL-10, (C) TNF-α, (D) IFN-γ, (E) KC and (F) MIP-2 were measured using Bio-Rad Bio-Plex assay. n = 6–8 in each group and P<0.05. *significantly different as compared to burn injury + vehicle treated group (B+V); #significantly different from burn injury + IL-15 SA treated group (B+IL-15); and $significantly different from burn injury + IL-15 SA treated infected group (B+IL-15+I).

As shown in Fig 8, the levels of pro-inflammatory cytokines in plasma, including IL-6, TNF-α, IFN-γ, KC and MIP-2, were significantly increased in both the vehicle and IL-15 SA treated infected groups, as compared to uninfected mice. Treatment with IL-15 SA further augmented cytokine production in this acute infection model (Fig 8). The levels of anti-inflammatory cytokine IL-10 were also significantly increased after infection and were amplified by IL-15 SA treatment.

## Discussion

Infection is the most common cause of death in burn patients that survive the initial burn trauma and a common cause of prolonged hospitalization [52]. Factors that predispose burned subjects to infection include loss of the skin barrier and, potentially, immunologic dysfunction. Our results show that combined cutaneous burn injury and infection with *Pseudomonas aeruginosa* leads to systemic dissemination of bacteria, liver and kidney injury, and systemic inflammation. Burn and infection caused a remarkable decline in circulating lymphocyte counts and a decrease in spleen CD4^+^ and CD8^+^ T cells on day 2 after infection. Treatment with IL-15 SA did not improve bacterial clearance or survival in this model. In fact, treatment with IL-15 SA caused a trend toward worsened liver and kidney injury in the wound infection model and significantly enhanced systemic cytokine production in the burn and systemic *Pseudomonas* challenge infection model.

Previous studies demonstrated T cell dysfunction and low absolute lymphocyte counts contribute to increased mortality in non-burned septic patients [8,19,53]. Sepsis is known to cause significant apoptosis-mediated death of monocytes, T, B, dendritic, and NK cells, which is postulated to cause immunosuppression and increased susceptibility to infection [54,55]. However, the effect of burn injury and infection on lymphocyte numbers and function has not been extensively studied. Previous studies using a mouse model of scald burn injury showed increased apoptotic cells in lymphoid tissues such as thymus, spleen and intestinal Peyer’s patches as early as 6 hours after burn injury [56,57]. Our study shows that burn injury causes a significant decline in circulating absolute lymphocyte cell counts as well as total and memory T cell numbers in the spleen. Superimposing infection upon burn injury caused further depletion of circulating lymphocytes and sustained the loss of splenic T cells. Thus, burn injury, especially in combination with infection, causes significant declines in peripheral and central lymphocytes. However, it is not clear whether lymphocyte depletion enhances susceptibility to infection after burn injury.

Treatments aimed at augmenting adaptive immune functions may decrease the incidence and severity of infection after burn injury. A previous study showed that IL-15 SA treatment can reverse lymphocyte dysfunction and improve survival in the cecal ligation and puncture (CLP) murine model of sepsis and in a model of *Pseudomonas* pneumonia [20]. IL-15 is known to have potent anti-apoptotic activity and stimulate the expansion and activation of T cells, especially memory CD8^+^ T (mCD8^+^ T), NKT and NK cells [30,50]. IL-15 super agonist (IL-15 SA) was employed in our study because of its prolonged half-life and enhanced potency as compared to IL-15 alone [41]. A recent study from our laboratory demonstrated that dosing with 2 μg IL-15 SA for 4 days expands mCD8^+^ T, NKT and NK cells. However, IL-15 SA in that regimen also caused liver injury and cachexia in the mouse, which was mediated by NK cells and dependent on production of interferon γ (IFNγ) [50]. Therefore, we limited the dose of IL-15 SA to 1 μg on alternate days after wound infection to minimize toxicity and because three doses of IL-15 SA led to a robust expansion of CD4^+^ and CD8^+^ T, NK and NKT cells in spleen and liver at day 4 post-burn injury. In addition to inducing lymphocyte expansion, IL-15 SA treatment also caused significant activation of CD4^+^T, CD8^+^T and NK cells as reflected by increased CD69 expression, in the spleen and liver. The augmented activation of lymphocytes might have had minor deleterious effects as indicated by marginally increased liver enzymes and increased cytokine production in mice receiving IL-15 SA treatment.

IL-15 SA treatment did not impact blood absolute lymphocyte counts after burn injury or burn plus infection, but increased CD4^+^T, CD8^+^T, NK, NKT and B lymphocytes in both spleen and liver tissue Sepsis is known to cause significant apoptosis-mediated death of monocytes, T, B, dendritic, and NK cells, which is postulated to cause immunosuppression and increased susceptibility to infection [54,55].of burned mice. However, all lymphocyte populations in spleen and liver tissue declined markedly after infection in IL-15 SA-treated mice and were only marginally greater than in vehicle-treated infected mice. Thus, treatment with IL-15 SA did not provide a sustained elevation in lymphocyte numbers after infection, which was reflected in its inability to augment antimicrobial immunity in our model. Treatment with IL-15 SA did not alter bacterial clearance in either the burn wound or systemic infection models nor did it effect neutrophil recruitment to the site of infection in mice receiving intraperitoneal *Pseudomonas* challenge. Innate immune cells, including neutrophils, monocytes and macrophages, form the first line of defense against bacterial infection and are required for rapid elimination of bacterial pathogens [58]. IL-15 is also known to prevent the apoptotic depletion of innate immune cells including dendritic and NK cells during sepsis [20]. To test if IL-15 might facilitate the innate immune cell recruitment and bacterial clearance, we employed an intraperitoneal *Pseudomonas aeruginosa* infection model. As shown in Fig 6, infection caused a significant decline in intraperitoneal macrophages (F4/80^+^Ly6G^-^ cells) and increase in neutrophils (Ly6G^+^F4/80^-^ cells). IL-15 failed to modify either macrophage or neutrophil populations in the peritoneal cavity. As a result, all infected mice developed severe infections and associated hypothermia. Our results are somewhat different than those reported by Inoue *et al* in which IL-15 SA was tested in models of CLP-induced sepsis and *Pseudomonas* pneumonia [20]. Both our study and theirs showed the ability of IL-15 SA to expand lymphocyte populations and to augment cytokine production during infection. However, we were unable to show improvements in survival or bacterial clearance in our model. The main difference between the studies is the presence of burn injury, which was a major component of our models. Thus, the presence of burn injury might significantly change the efficacy of IL-15 SA as an immunotherapeutic agent. It is also worthwhile to mention that, Inoue *et al*., employed CD-1 mice strain as opposed to BALB/c mice in our studies, which might also be one of the reasons for the observed differences in survival [20].

Burn injury is also known to induce a state of inflammation with respect to increased pro-inflammatory cytokine production [59]. In our study, burn injury alone caused a significant increase in the classic pro-inflammatory cytokine IL-6 on day 4 following injury. Infection led to a further increase in the levels of plasma pro-inflammatory cytokines including IL-6, TNF-α, KC and MIP-2. IL-15 SA treatment did not induce a significant upregulation of pro-inflammatory cytokines in mice receiving burn injury alone. Moreover, IL-15 treatment did not significantly augment pro-inflammatory cytokine production in mice with burn wound infection. As seen in Fig 6, there was a trend towards increased plasma KC and MIP-2 after burn wound infection in both vehicle and IL-15 SA treated groups, as compared to the sham group. But none of these changes were statistically significant. This is likely due to wide variability in plasma cytokines at the day 2 measurement point in the wound infection model. The dissemination of bacteria from wound to blood is variable in the wound infection model. Plasma cytokine concentrations closely parallel blood bacterial counts. Thus, there is variability in both blood bacterial counts and plasma cytokines in this model. Nevertheless, there was a strong trend toward increased plasma KC and MIP-2 concentrations after burn wound infection that was potentiated by IL-15 SA treatment. While the increased KC and MIP-2 levels might increase PMN numbers, our data shows that PMN’s actually decline in the blood after wound infection as compared to the sham group in both vehicle- and IL-15 SA-treated mice. This is likely due to the active migration of PMN’s to the site of burn wound infection.

We used the intraperitoneal infection model to overcome the variability associated with the wound infection model. The intraperitoneal challenge model provides for consistent dissemination of bacteria and more uniform induction of systemic cytokine production. The levels of plasma IL-6, TNF-α, IFN-γ and MIP-2 were significantly higher in infected mice and in the IL-15 SA-treated mice receiving intraperitoneal *Pseudomonas* infection as compared to vehicle treated infected mice. This implies that IL-15 SA exacerbates the pro-inflammatory response following burn injury and *Pseudomonas aeruginosa* sepsis. Although not statistically significant, there was a trend towards higher bacterial CFUs in the IL-15 SA treated group (15.5x10^6^ CFU’s) as compared to vehicle treatment (11.8x10^6^ CFU’s) after intraperitoneal *Pseudomonas* challenge, implying a relatively impaired bacterial clearance. That finding paralleled lower neutrophil counts in the peritoneal cavity of IL-15 SA-treated mice after infection. Infection also caused a significant increase in the levels of plasma KC and MIP-2 and MIP-2 concentrations were higher in IL-15 SA-treated mice than in vehicle controls. Based on these observations, it is plausible that IL-15 SA treatment actually impaired PMN recruitment and bacterial clearance as compared to vehicle treated group. This may be due to the higher plasma chemokine concentrations in the IL-15 SA-treated group, which can wash out plasma to tissue chemokine gradients and result in impaired mobilization of neutrophils to sites of infection [60,61].

## Conclusion

Our study demonstrates that burn injury reduces absolute lymphocyte numbers, and shows that burned mice are highly susceptible to secondary burn-wound infection. However, while IL-15 SA expands and activates T, B, NK, and NKT cells in burn injured mice, it fails to improve survival after a *Pseudomonas aeruginosa* burn wound infection. The potential reasons include failure of IL-15 SA treatment to enhance clearance of the systemic bacterial load and possible potentiation of organ injury due augmentation of pro-inflammatory cytokine production. Based on results of this study, one might hypothesize that impaired adaptive immune responses might not be a significant contributing factor towards susceptibility to infection following burn injury. However, IL-15 SA predominantly expands NK, NKT, and mCD8+ lymphocytes, whereas preferential expansion of naïve and effector CD4 and CD8 T-lymphocyte populations, with therapies such as IL-7 [21,62], may provide more effective resistance to infection. Further work is needed to determine the impact of adaptive immune impairment after burn and selective approaches to improve adaptive immunity in burned subjects merit further investigation. Previous studies from our laboratory, and others, have shown that the dendritic cell hemopoietic factor Flt3 ligand as well as TLR4 ligand monophosphoryl lipid A are very effective in augmenting antimicrobial immunity in burned mice [63–66]. Thus, approaches that improve innate immune function, in addition to adaptive immune function, may be a more useful strategy to decrease mortality in burn-injured patients.
